# DELIRIUM-LIKE BEHAVIORAL SYMPTOMATOLOGY IN COMMUNITY-DWELLING OLDER ADULTS WITH DEMENTIA

**DOI:** 10.1093/geroni/igac059.1818

**Published:** 2022-12-20

**Authors:** Jasmine Vickers, Danny Wang, Carolyn Pickering

**Affiliations:** University of Alabama at Birmingham, Birmingham, Alabama, United States; The University of Alabama at Birmingham, Birmingham, Alabama, United States; The University of Alabama at Birmingham, Birmingham, Alabama, United States

## Abstract

Delirium, a sudden and acute confusional state, is known to be more prevalent and deleterious in older adults with dementia; yet the literature on estimates of delirium in community-dwelling older adults with dementia is scarce. The aim of this study was to determine the patterns and frequency of delirium-like symptoms in community-dwelling older adults with dementia, as reported by their caregivers (n=50) in a 21-day diary study (n=1389 diaries). Caregiver reports of severity scores of care-recipient behaviors that were one standard deviation above the mean were considered outliers, representing a sudden and acute change. Caregivers that reported outlier scores for two consecutive days were considered delirium-like symptoms. Descriptive statistics were used to determine the number of caregivers who reported delirium-like symptoms and the percentage of days with symptoms present. Additionally, chi-square tests and Independent samples T-test were used to determine the associations between delirium-like symptoms and participant characteristics. Caregivers were predominately White/Caucasian (89%), female (94%), and a son/daughter (60%) of the care-recipient. Caregivers had an average age of 52.6 years and were caregiving an average of 3.9 years. The care-recipients had dementia for an average of 4.3 years. Caregivers reported delirium-like symptoms for 12(24%) care-recipients and this rate was consistent with previous research findings. Caregivers reported delirium-like symptoms, on average, for 6.9 days (33%) of the 21-day diary period. More research is needed to understand the occurrence and patterns of delirium in community-dwelling older adults with dementia to improve home care services, outpatient services, and support for caregivers.

